# Neuraxial labor analgesia use among racial and ethnic groups in the United States, 2016–2022

**DOI:** 10.1002/pmf2.70127

**Published:** 2025-10-08

**Authors:** Eva Martinez, Sara Siadat, Emily Stockert, Brian T. Bateman, Alexander J. Butwick, Stephanie A. Leonard

**Affiliations:** ^1^ Department of Anesthesiology Perioperative and Pain Medicine Stanford University School of Medicine Stanford California USA; ^2^ Department of Anesthesia and Perioperative Care University of California San Francisco San Francisco California USA; ^3^ Department of Obstetrics and Gynecology Stanford University School of Medicine Stanford California USA

**Keywords:** analgesia, disparities, epidural, ethnic and racial minorities, healthcare, obstetrical

## Abstract

**Introduction:**

Neuraxial analgesia is the most effective modality for pain relief during labor; yet utilization varies considerably among racial and ethnic groups in the United States—representing a health disparity. Little is known about how maternal and obstetric characteristics may contribute to these differences and whether variation exists among a larger number of racial and ethnic groups. The objective of our study was to evaluate differences in neuraxial labor analgesia use among racial and ethnic groups in the United States.

**Methods:**

We analyzed vital statistics data from in‐hospital spontaneous vaginal births of singletons during 2016 to 2022 in the United States (*N* = 26,345,765). Race and Hispanic ethnicity were considered as social constructs, measured by self‐report, and combined into seven racial and ethnic groups for analysis. The outcome of interest was neuraxial labor analgesia use. We conducted sequentially adjusted multivariable modified Poisson regression models to estimate relative risks (RRs) with 95% confidence intervals (CIs) for associations between racial and ethnic groups and neuraxial labor analgesia. We first adjusted for delivery year and maternal characteristics: age, body mass index, insurance status, and educational background. We then additionally adjusted for obstetric characteristics: timing of prenatal care initiation, gestational age at delivery, and obstetric history (prior live birth and prior cesarean birth). We further descriptively assessed variation in utilization among Asian, Native Hawaiian or Other Pacific Islander (NHOPI), and Hispanic subgroups.

**Results:**

The rate of neuraxial labor analgesia use was 74% among 15,373,550 spontaneous vaginal, singleton births in the United States. White individuals had the highest rate (78%), and NHOPI and American Indian and Alaska Native (AI/AN) individuals had the lowest rates (58% and 61%, respectively) of use. After adjustment for covariates, the RR of using neuraxial labor analgesia was lowest in NHOPI individuals (0.78; 95% CI, 0.77–0.79) and AI/AN individuals (0.82; 95% CI, 0.82–0.82) compared with White individuals. The fully adjusted RRs were 0.93 (95% CI, 0.93–0.93) for Hispanic individuals, 0.98 (95% CI, 0.98–0.98) for Asian individuals, 0.96 (95% CI, 0.96–0.96) for Black individuals, and 0.97 (95% CI, 0.97–0.98) for multiracial individuals. In a secondary analysis, utilization ranged from 70% to 82% among Asian subgroups, 60% to 69% among NHOPI subgroups, and 64% to 81% among Hispanic subgroups.

**Conclusions:**

Neuraxial labor analgesia use in the United States was lowest among NHOPI and AI/AN individuals, independent of measured maternal and obstetric characteristics. Efforts are needed to understand and address disparities in contemporary practice with a particular focus on healthcare access and NHOPI and AI/AN communities.

## 1 INTRODUCTION

Neuraxial analgesia use has been shown to be the most effective modality for pain relief during labor [1]. The American College of Obstetricians and Gynecologists and the American Society of Anesthesiologists (ASA) both promote the use of neuraxial analgesia during labor as safe and effective [2, 3]. Despite this, substantial racial and ethnic disparities in the use of neuraxial labor analgesia exist [4, 5]. As of 2022, the use of neuraxial labor analgesia in the United States by nulliparous individuals with vaginal, singleton live birth ranges from 69% among Native Hawaiian or Other Pacific Islander (NHOPI) individuals to 86% among Asian and White individuals [6]. Yet, little is known about how maternal and obstetric characteristics may contribute to these differences and whether variation exists among a larger number of racial and ethnic subpopulations. The ASA has stated that understanding racial and ethnic disparities in neuraxial labor analgesia is essential [2], as these racial and ethnic groups are social constructs and lower usage in marginalized groups represents health disparities [2]. We conducted this study in response to this evidence gap, using contemporary national data to evaluate differences in neuraxial labor analgesia use among racial and ethnic groups in the United States.

## 2 MATERIALS AND METHODS

### 2.1 Study design

This was a retrospective, cross‐sectional study. As such, we followed the Strengthening the Reporting of Observational Studies in Epidemiology (STROBE) reporting guidelines. All data used are publicly available from the Centers for Diseases Control and Prevention (CDC) [7]. This study did not involve patient‐identifiable data or research on human subjects. As such, it did not meet requirements for Institutional Review Board approval.

### 2.2 US birth certificate database

The investigators analyzed US vital statistics data provided by the National Vital Statistics System (National Center for Health Statistics, Hyattsville, Maryland; Centers for Disease Control and Prevention, Atlanta, Georgia). The Birth File is a census of all recorded live births in the United States and contains detailed information collected on birth certificate forms about the mother, father, pregnancy, labor, and delivery. These data are provided through vital registration systems that are maintained and operated by states and territories where the original certificates are filed. The National Center for Health Statistics, in collaboration with state vital statistics offices, revised the US Standard Certificates of Live Birth and Death to include the use of epidural or spinal anesthesia during labor in 2003. The implementation of the 2003 revisions was fully complete across all 50 US states in 2016. Thus, we chose to study the years 2016 and on for the uniformity of data tracking on epidural usage across the country. The study data are publicly available online [7].

### 2.3 Study population

The study sample included live births in the United States from January 2016 to December 2022. We restricted to spontaneous, singleton, in‐hospital, vaginal births for the primary analysis due to concerns about confounding by multifetal gestation and about the influence of neuraxial placement for intrapartum cesarean births and operative vaginal births (vacuum and forceps). We examined intrapartum cesarean births (cesarean birth following a trial of labor) in a secondary analysis. Individuals were excluded from the study sample if they were missing information on variables used for sample selection or in the regression analysis: live birth order, racial and ethnic group, final route and method of delivery, neuraxial labor analgesia use, body mass index (BMI), payment source for delivery, maternal education, gestational age, prior cesarean delivery, and month of pregnancy when prenatal care was initiated. For a secondary analysis, we identified and restricted to intrapartum cesarean births. We identified these as births with a trial of labor indicated and cesarean birth as the final route of delivery.

### 2.4 Study variables

The exposure or predictor variable of interest was race and ethnicity, which is a social construct and characteristic that is self‐reported with high validity in birth certificate data [8]. For the main analysis, we used seven racial and ethnic categories following the US Office of Management and Budget standards for maintaining, collecting, and presenting federal data on race and ethnicity [9]. These categories included: American Indian or Alaska Native (AI/AN), Asian, Black or African American, NHOPI, Hispanic, White, and more than one race. Individuals of any racial identity who identified as Hispanic, Latino, or Spanish ethnicity were classified as Hispanic. To identify variation within these categories using available data, we additionally disaggregated Asian (Asian Indian, Chinese, Filipino, Japanese, Korean, Vietnamese, Other Asian), NHOPI (Guamanian, Hawaiian, Samoan, Other Pacific Islander), and Hispanic (Central or South American, Cuban, Mexican, Puerto Rican, Other or Unknown Hispanic).

The outcome of interest was neuraxial labor analgesia use. Information on birth certificates is collected for each birth by the birthing facility based on clinical records and maternal surveys. Each birth certificate contains a checkbox to delineate whether the patient received “epidural or spinal anesthesia during labor.” This checkbox was found to have high sensitivity in analysis comparison against medical records for 1095 births in two states (96% and 85%, respectively) [10]. There are no further specifications in the birth certificate about the type of neuraxial used (epidural, spinal, combined spinal epidural, dural puncture epidural) or other analgesic modalities (e.g., intravenous opioids, inhaled nitrous oxide).

Maternal and obstetric characteristics were identified and used as covariates in the analysis. Maternal characteristics included: age, BMI, payment source for delivery, and educational background. Obstetric characteristics included: timing of prenatal care initiation, gestational age at delivery, and obstetric history (nulliparous—no prior live birth, multiparous without prior cesarean birth, or multiparous with prior cesarean birth).

### 2.5 Statistical analysis

We first calculated the crude incidence of neuraxial labor analgesia use overall and stratified by race and ethnicity, maternal characteristics, and obstetric characteristics. We then fit sequentially adjusted multivariable modified Poisson regression models to estimate relative risks (RRs) and 95% confidence intervals (CIs) for racial and ethnic differences in labor neuraxial analgesia use. Unadjusted estimated RRs of labor neuraxial use were preliminarily computed. The first adjusted multivariable model adjusted for delivery year and maternal characteristics. The second adjusted multivariable model (Model 3) additionally adjusted for obstetric characteristics.

We conducted two sets of secondary analyses. (1) We calculated the use of neuraxial labor analgesia among Asian, NHOPI, and Hispanic subgroups. (2) We conducted multivariable regression analyses among cesarean births following a trial of labor. The statistical analysis was performed using R version 4.4.1 [11].

## 3 RESULTS

**FIGURE 1:**
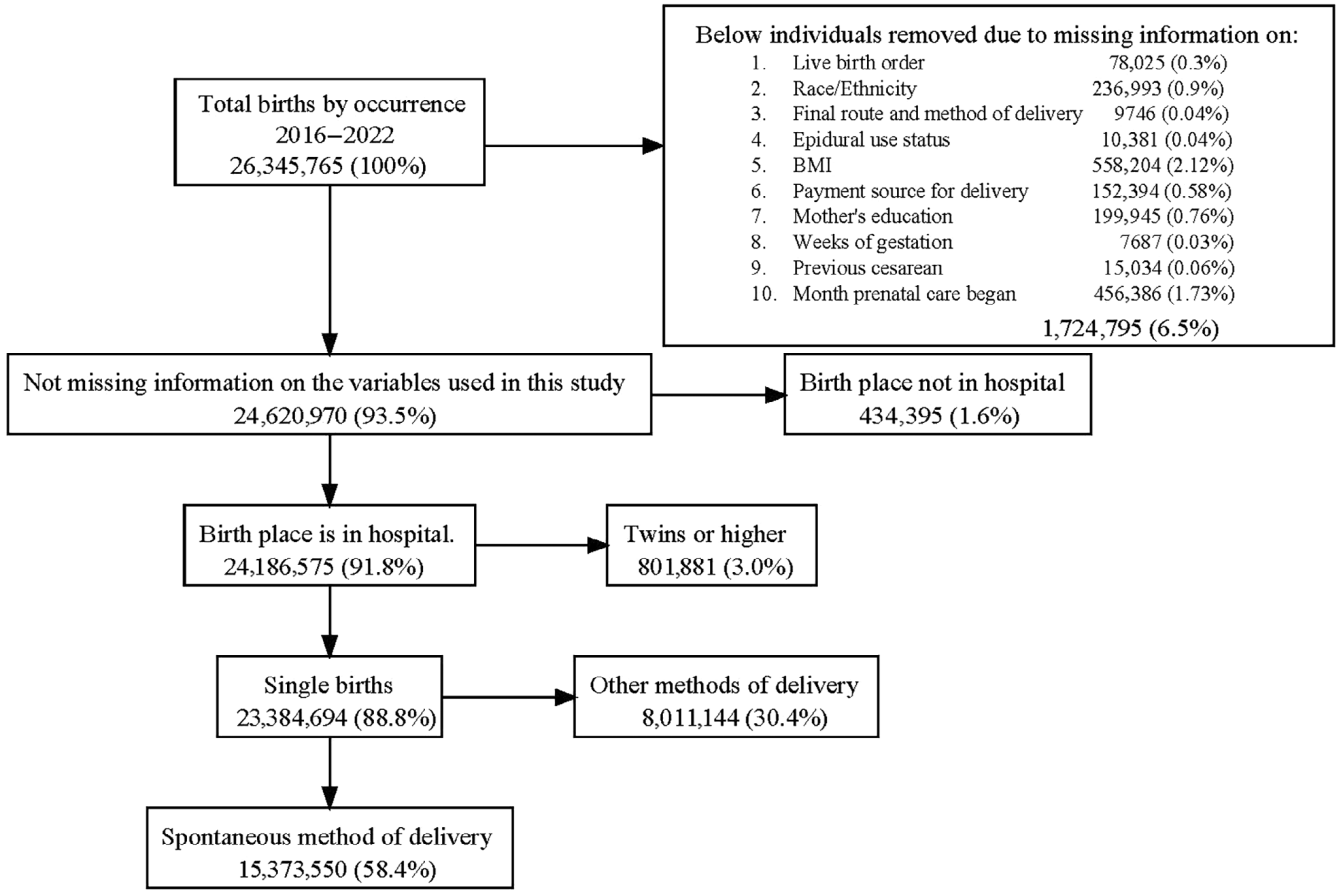
Sample selection figure, US live births, 2016–2022 BMI, body mass index.

Between 2016 and 2022, a total of 15,373,550 singleton, spontaneous vaginal births across the United States met study inclusion criteria (Figure 1). Neuraxial labor analgesia use was reported for 11,422,220 births, yielding an overall usage rate of 74%. Among the seven racial and ethnic groups, White individuals had the highest crude rate of neuraxial labor analgesia use of 78%, with Asian (75%) and multiracial individuals (75%) tied as the second highest (Table 1). AI/AN individuals (61%) and NHOPI individuals (58%) had the lowest labor neuraxial analgesia rates. Rates of neuraxial labor analgesia were also lower in individuals who had Medicaid insurance compared with private health insurance (71% vs. 78%), lower educational attainment (65% in individuals with a high school degree or less vs. 78% in individuals with a college degree or higher), preterm birth (69% vs. 75%), no prenatal care (60% vs. 76% with care initiated in first trimester), and multiparous individuals without a prior cesarean delivery (69% vs. 82% in nulliparous individuals).

**TABLE 1:** Characteristics of individuals in the study sample, spontaneous vaginal births, the United States, 2016–2022.

Characteristic	Total population *N* = 15,373,550 *n* (row %)	Neuraxial labor analgesia used *N* = 11,422,220 *n* (row %)	Neuraxial labor analgesia not used *N* = 3,951,330 *n* (row %)
Racial and ethnic group			
Hispanic/Latino	3,811,963 (100)	2,628,365 (69)	1,183,598 (31)
AI/AN	123,600 (100)	75,695 (61)	47,905 (39)
Asian	935,830 (100)	699,565 (75)	236,265 (25)
Black	2,076,510 (100)	1,502,679 (72)	573,831 (28)
NHOPI	37,845 (100)	21,798 (58)	16,047 (42)
Multiracial	357,652 (100)	269,449 (75)	88,203 (25)
White	8,030,150 (100)	6,224,669 (78)	1,805,481 (22)
Maternal age			
Less than 35 years old	12,953,183 (100)	9,691,785 (75)	3,261,398 (25)
35 years or older	2,420,367 (100)	1,730,435 (71)	689,932 (29)
Body mass index			
<30 kg/m^2^	11,505,206 (100)	8,497,129 (74)	3,008,077 (26)
≥30 kg/m^2^	3,868,344 (100)	2,925,091 (76)	943,253 (24)
Payment source for birth			
Medicaid	6,582,366 (100)	4,684,696 (71)	1,897,670 (29)
Private insurance	7,694,669 (100)	5,988,377 (78)	1,706,292 (22)
Self‐pay	514,615 (100)	317,910 (62)	196,705 (38)
Other	581,900 (100)	431,237 (74)	150,663 (26)
Educational attainment			
High school degree or less	1,954,026 (100)	1,265,804 (65)	688,222 (35)
High school degree or equivalent	4,088,651 (100)	2,988,638 (73)	1,100,013 (27)
Some college	4,250,565 (100)	3,222,603 (76)	1,027,962 (24)
College degree or higher	5,080,308 (100)	3,945,175 (78)	1,135,133 (22)
Gestational age			
Preterm (<37 weeks gestation)	997,288 (100)	685,444 (69)	311,844 (31)
Term (≥37 weeks gestation)	14,376,262 (100)	10,736,776 (75)	3,639,486 (25)
Initiation of prenatal care			
First trimester	11,936,572 (100)	9,027,302 (76)	2,909,270 (24)
Second trimester	2,494,943 (100)	1,760,839 (71)	734,104 (29)
Third trimester	680,582 (100)	477,820 (70)	202,762 (30)
No prenatal care	261,453 (100)	156,259 (60)	105,194 (40)
Obstetric history			
Nulliparous (no prior live birth)	5,908,524 (100)	4,837,736 (82)	1,070,788 (18)
Multiparous without prior cesarean birth	9,010,261 (100)	6,244,363 (69)	2,765,898 (31)
Multiparous with prior cesarean birth	454,765 (100)	340,121 (75)	114,644 (25)

Abbreviations: AI/AN, American Indian or Alaska Native; NHOPI, Native Hawaiian or Other Pacific Islander.

**FIGURE 2:**
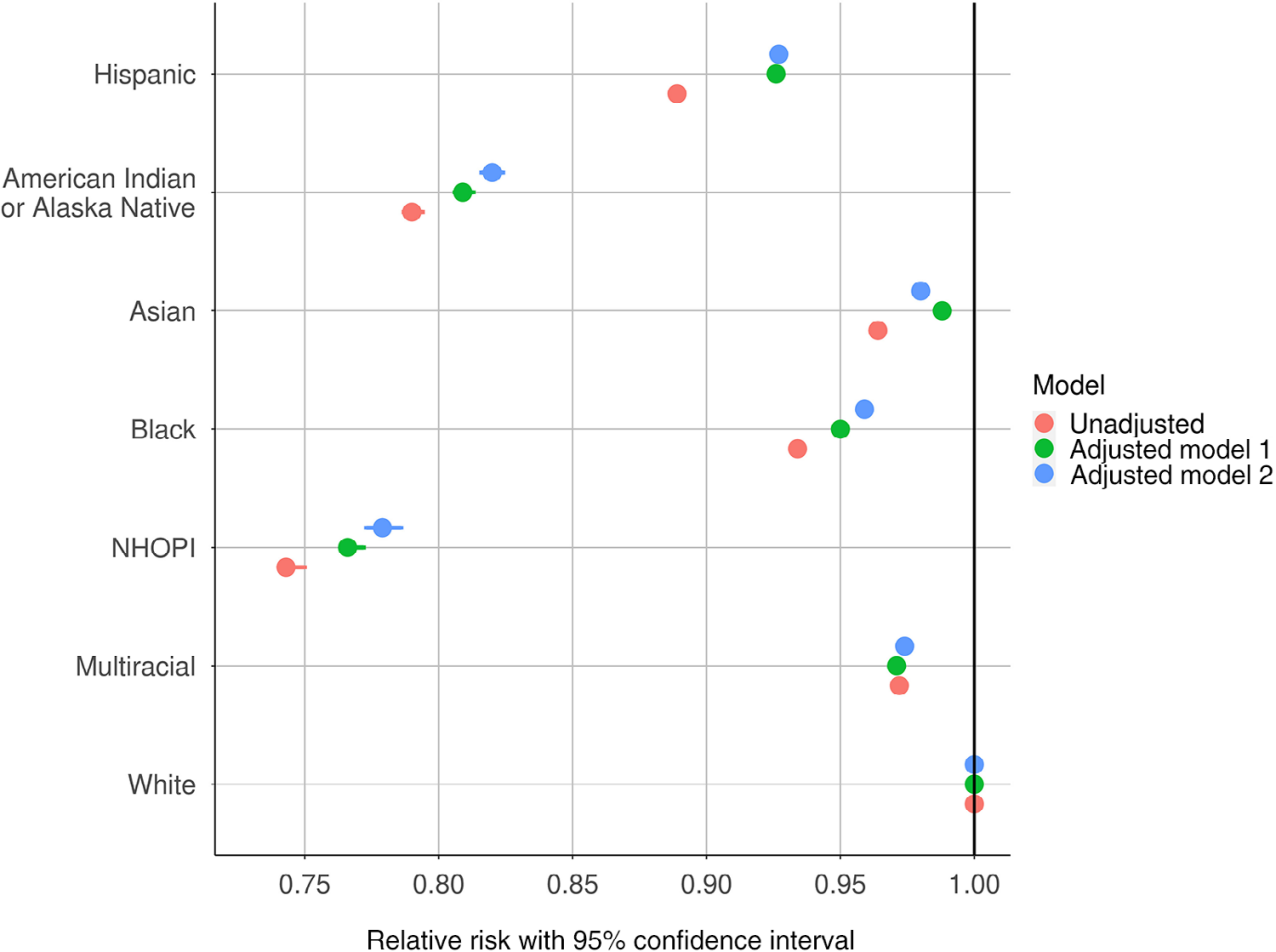
Differences in neuraxial labor anesthesia use during labor for spontaneous vaginal birth among racial and ethnic groups, the United States, 2016–2022. Adjusted Model 1 includes year and maternal characteristics. Adjusted Model 2 additionally included obstetric characteristics. NHOPI, Native Hawaiian and Other Pacific Islander.

Consistent with the crude results, unadjusted regression modeling showed that every non‐White racial and ethnic group had lower crude rates of neuraxial analgesia compared with White individuals (Table 2, Figure 2). Statistical adjustment for maternal and obstetric characteristics had a minimal effect on the estimated RRs and CIs. After full adjustment, the RR of using labor neuraxial analgesia was lowest in NHOPI individuals (0.78; 95% CI, 0.77–0.79) and AI/AN individuals (0.82; 95% CI, 0.82–0.82) compared with White individuals. The fully adjusted RRs were 0.93 (95% CI, 0.93–0.93) for Hispanic individuals, 0.98 (95% CI, 0.98–0.98) for Asian individuals, 0.96 (95% CI, 0.96–0.96) for Black individuals, and 0.97 (95% CI, 0.97–0.98) for multiracial individuals.

**TABLE 2:** Differences between racial and ethnic groups in neuraxial labor analgesia use for spontaneous vaginal births, the United States, 2016–2022.

	Unadjusted RR (95% CI)	Adjusted for maternal characteristics^a^ + year RR (95% CI)	Adjusted for maternal + obstetric characteristics^b^ + year RR (95% CI)
Racial and ethnic group			
Hispanic	0.89 (0.89–0.89)	0.93 (0.93–0.93)	0.93 (0.93–0.93)
AI/AN	0.79 (0.79–0.79)	0.81 (0.81–0.81)	0.82 (0.82–0.82)
Asian	0.96 (0.96–0.97)	0.99 (0.99–0.99)	0.98 (0.98–0.98)
Black	0.93 (0.93–0.93)	0.95 (0.95–0.95)	0.96 (0.96–0.96)
NHOPI	0.74 (0.74–0.75)	0.77 (0.76–0.77)	0.78 (0.77–0.79)
Multiracial	0.97 (0.97–0.97)	0.97 (0.97–0.97)	0.97 (0.97–0.98)
White	Reference	Reference	Reference

Abbreviations: AI/AN, American Indian or Alaska Native; CI, confidence interval; NHOPI, Native Hawaiian and Other Pacific Islander; RR, relative risk.

^a^ Age, body mass index, insurance status, and educational background.

^b^ Timing of prenatal care initiation, gestational age at delivery, and obstetric history.

**FIGURE 3:**
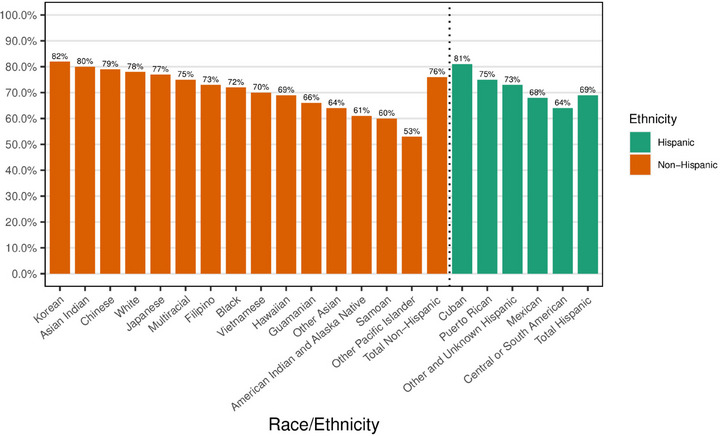
Neuraxial labor analgesia use among all available racial and ethnic groups, spontaneous vaginal births, the United States, 2016–2022.

In a secondary descriptive analysis, we found variation in neuraxial labor analgesia use among Asian, NHOPI, and Hispanic subgroups (Figure 3). Among seven Asian subgroups, neuraxial labor analgesia use ranged from 70% in Vietnamese to 82% in Korean. Among four NHOPI subgroups, use ranged from 60% in Samoan to 69% in Hawaiian. Among five Hispanic subgroups, use ranged from 64% in Central or South American to 81% in Cuban. Cuban individuals were the only Hispanic subgroup with higher neuraxial labor analgesia use than White individuals.

In a secondary analysis among intrapartum cesarean births, we included 2,054,859 births (7.8% of total live births). Among these births, neuraxial labor analgesia ranged from 84% in AI/AN individuals to 94% in Asian individuals (Table 3). In the fully adjusted multivariable regression models, the relative risk of neuraxial labor analgesia use was lowest in AI/AN individuals (0.94; 95% CI, 0.93–0.95) and NHOPI individuals (0.97; 95% CI, 0.96–0.98), compared with White individuals. In contrast, the RR of using neuraxial labor analgesia was higher in Hispanic, Asian, and Black birthing individuals compared with White individuals after adjustment for covariates.

**TABLE 3:** Differences between racial and ethnic groups in neuraxial labor analgesia use for intrapartum cesarean births, the United States, 2016–2022.

	Neuraxial labor analgesia use *N* (row %)	Unadjusted RR (95% CI)	Adjusted for maternal characteristics^a^ + year RR (95% CI)	Adjusted for maternal + obstetric characteristics^b^ + year RR (95% CI)
Racial and ethnic group			
Hispanic	399,221 (92)	1.00 (1.01–1.01)	1.02 (1.02–1.02)	1.02 (1.02–1.02)
AI/AN	12,063 (84)	0.92 (0.92–0.93)	0.94 (0.93–0.94)	0.94 (0.93–0.95)
Asian	128,846 (94)	1.03 (1.02–1.03)	1.02 (1.02–1.02)	1.02 (1.02–1.02)
Black	318,075 (91)	0.99 (0.99–0.99)	1.01 (1.01–1.01)	1.01 (1.01–1.01)
NHOPI	4320 (87)	0.95 (0.94–0.96)	0.96 (0.95–0.97)	0.97 (0.96–0.98)
Multiracial	44,587 (91)	1.00 (0.99–1.00)	1.01 (1.00–1.01)	1.01 (1.00–1.01)
White	976,298 (91)	Reference	Reference	Reference

Abbreviations: AI/AN, American Indian or Alaska Native; CI, confidence interval; NHOPI, Native Hawaiian and Other Pacific Islander; RR, relative risk.

^a^ Age, body mass index, insurance status, and educational background.

^b^ Timing of prenatal care initiation, gestational age at delivery, and obstetric history.

## 4 DISCUSSION

Among seven major racial and ethnic groups in the United States, the lowest rate of neuraxial labor analgesia use was among AI/AIN and NHOPI individuals. These groups also had the lowest rate of neuraxial use in a secondary analysis among intrapartum cesarean births. These findings persisted after adjusting for maternal and obstetric characteristics. Further disaggregation of racial and ethnic groups revealed variation within Asian, NHOPI, and Hispanic groups. Reduced use of neuraxial labor analgesia may imply a continued barrier to access of high‐quality obstetric anesthesiology care [2].

Prior studies have shown that racial and ethnic minority groups face a variety of structural barriers and racism in healthcare, including anesthesiology [12, 13, 14]. In obstetrics, racially minoritized individuals (those belonging to racial and ethnic minority groups) are more likely to experience severe postpartum pain [15] and less likely to receive neuraxial labor analgesia [4, 16, 17, 18, 19]. Our study builds on this prior body of evidence, assessing variation in neuraxial labor analgesia use among racial and ethnic groups defined with greater granularity than have been examined in previous research. Each racial and ethnic group has unique characteristics that could affect their anesthesiology care as this relates to cultural, financial, educational, or language‐based barriers.

Our finding of the lowest neuraxial labor analgesia usage in AI/AN and NHOPI individuals is consistent with results from other obstetrical health disparity studies. Labor and delivery complications, including severe maternal and neonatal morbidity and mortality, affect AI/AN and NHOPI individuals at high rates compared with other racial and ethnic groups [20, 21]. They also have the highest rates of late or no prenatal care in the United States, at 12.5% and 21.0%, respectively [22]. Further, AI/AN individuals are the most likely to give birth at rural hospitals [20], which have lower neuraxial labor analgesia use due to barriers such as limited availability of anesthesiologists [17]. However, in reporting of healthcare differences between racial and ethnic groups, AI/AN and NHOPI representation has been scarce and largely grouped as “other” racial and ethnic category or as Asian/Pacific Islander, if mentioned at all [19, 23]. Our study underscores the pitfalls of grouping Asian and Pacific Islander, as the highest rate of neuraxial labor analgesia (79%–80%) was among Asian subgroups (Korean, Asian Indian, Chinese) and the lowest rate (53%–60%) among NHOPI subgroups (Samoan and Other Pacific Islander). Further, AI/AN individuals represent an important birthing population in the United States with unique obstetric healthcare challenges and opportunities for improved care [20].

Our secondary analysis of individuals who underwent intrapartum cesarean deliveries revealed a similar pattern of disparities as for spontaneous vaginal births, but smaller in magnitude. After adjustment for maternal and obstetric characteristics, only AI/AN and NHOPI had lower rates than White. Further, Hispanic, Asian, and Black individuals had modestly higher use of neuraxial labor analgesia compared with White individuals after covariate adjustment. Prior studies have found that certain minoritized racial and ethnic groups tend to receive general anesthesia at higher rates than White individuals for cesarean births [18, 23, 24]. Our data set did not allow us to glean the temporal sequence of events that led to a cesarean birth, or whether this patient population may have received neuraxial labor analgesia preemptively based on risk factors for operative delivery (e.g., arrest of labor, non‐reassuring fetal heart tracing, or large for gestational age fetus). One possible explanation for our findings is that minoritized individuals with risk factors for operative deliveries could be exposed to neuraxial labor analgesia more so than minoritized individuals without the same risk factor profile.

Limitations of this study should be noted. Geographical, hospital, provider, and social factors were not available and likely contribute to differences in neuraxial labor analgesia use among racial and ethnic groups. Such differences could be affected by variation in hospital factors, including the number of triage and labor rooms, in‐house obstetrician and anesthesiologist on call, whether anesthesiologists are obstetric fellowship trained, and the level of maternity care. Additionally, social determinants, including language, social support, and experience of racism, may affect racial and ethnic differences in neuraxial labor analgesia use. Further, the reason for neuraxial labor analgesia use or non‐use was unknown in our study. Measurement and analysis of such factors is an area of future research that is critical to understanding and addressing the racial and ethnic disparities observed in this retrospective study. It is also important to note that the birth certificate checkbox for neuraxial labor analgesia in a validation study had high sensitivity, but a high false discovery rate—reflecting potential misreporting when compared with medical records [10].

Our study also has several strengths. It provides a contemporary examination of racial and ethnic disparities in neuraxial labor analgesia use, including all recorded spontaneous, singleton vaginal births in the United States. We were also able to analyze an exhaustive database of birthing individuals by every racial and ethnic group adherent to federal standards, including those that have historically been especially underrepresented in health research. Race and ethnicity are self‐reported on the birth certificate and have been shown to have high validity [8, 25]. We were also able to measure and account for maternal and obstetric characteristics that are captured on birth certificate data with high validity [25].

## 5 CONCLUSION

Neuraxial labor analgesia use among all recorded spontaneous vaginal, singleton births in the United States from 2016 to 2022 varied between racial and ethnic groups with the lowest rates among AI/AN and NHOPI individuals, independent of measured maternal and obstetric characteristics. Rates were also lowest—to a lesser extent—for AI/AN and NHOPI in an analysis of intrapartum cesarean births. We further observed considerable heterogeneity in neuraxial labor analgesia use among Hispanic, Asian, and NHOPI subgroups. These findings suggest that a more detailed and careful assessment of specific communities is warranted to further understand and address their disparities in labor neuraxial use. Additional investigation into the mechanisms that are driving these disparities in neuraxial labor analgesia is needed with the goal of finding solutions at the geographical, hospital, provider, and patient levels.

## CONFLICT OF INTEREST STATEMENT

The authors declare no conflicts of interest.

## FUNDING INFORMATION

This research received no specific funding for this study.
